# Profiling of drought-responsive microRNA and mRNA in tomato using high-throughput sequencing

**DOI:** 10.1186/s12864-017-3869-1

**Published:** 2017-06-26

**Authors:** Minmin Liu, Huiyang Yu, Gangjun Zhao, Qiufeng Huang, Yongen Lu, Bo Ouyang

**Affiliations:** Key Laboratory of Horticultural Plant Biology (MOE), Huazhong Agricultural University, Wuhan, 430070 China

**Keywords:** Tomato, microRNA, RNA-Seq, Drought, Introgression line

## Abstract

**Background:**

Abiotic stresses cause severe loss of crop production. Among them, drought is one of the most frequent environmental stresses, which limits crop growth, development and productivity. Plant drought tolerance is fine-tuned by a complex gene regulatory network. Understanding the molecular regulation of this polygenic trait is crucial for the eventual success to improve plant yield and quality. Recent studies have demonstrated that microRNAs play critical roles in plant drought tolerance. However, little is known about the microRNA in drought response of the model plant tomato. Here, we described the profiling of drought-responsive microRNA and mRNA in tomato using high-throughput next-generation sequencing.

**Results:**

Drought stress was applied on the seedlings of M82, a drought-sensitive cultivated tomato genotype, and IL9–1, a drought-tolerant introgression line derived from the stress-resistant wild species *Solanum pennellii* LA0716 and M82. Under drought, IL9–1 performed superior than M82 regarding survival rate, H_2_O_2_ elimination and leaf turgor maintenance. A total of four small RNA and eight mRNA libraries were constructed and sequenced using Illumina sequencing technology. 105 conserved and 179 novel microRNAs were identified, among them, 54 and 98 were differentially expressed upon drought stress, respectively. The majority of the differentially-expressed conserved microRNAs was up-regulated in IL9–1 whereas down-regulated in M82. Under drought stress, 2714 and 1161 genes were found to be differentially expressed in M82 and IL9–1, respectively, and many of their homologues are involved in plant stress, such as genes encoding transcription factor and protein kinase. Various pathways involved in abiotic stress were revealed by Gene Ontology and pathway analysis. The mRNA sequencing results indicated that most of the target genes were regulated by their corresponding microRNAs, which suggested that microRNAs may play essential roles in the drought tolerance of tomato.

**Conclusion:**

In this study, numerous microRNAs and mRNAs involved in the drought response of tomato were identified using high-throughput sequencing, which will provide new insights into the complex regulatory network of plant adaption to drought stress. This work will also help to exploit new players functioning in plant drought-stress tolerance.

**Electronic supplementary material:**

The online version of this article (doi:10.1186/s12864-017-3869-1) contains supplementary material, which is available to authorized users.

## Background

Drought is the most severe abiotic stress affecting global agriculture, which seriously reduces crop yield and product quality [1]. Understanding the mechanisms underlying drought tolerance is critical for sustainable agriculture [2]. In the long-term evolution, plants have developed a series of protective mechanisms under drought condition, which function at the morphological, physiological, biochemical, cellular and molecular levels. Typical mechanisms include development of vigorous root system, formation of epidermal wax, regulation of stomatal conductance to reduce respiration, production of osmolytes, elimination of reactive oxygen species, and mobilization of stress-related hormones [3–6]. Each mechanism depends on the expression and regulation of a large number of genes.

To investigate the expression of these genes under stress condition, the “omic” approach has been extensively applied, which was further facilitated with the evolution of high-throughput sequencing technology. It appears to be a powerful tool to understand the molecular network under plant stress [7, 8]. For instance, transcriptomic technology has been successful to provide gene expression data upon abiotic stress in *Arabidopsis*, tomato, maize, and etc. [7, 9, 10]. This technology has also been applied in microRNAs (miRNAs) profiling.

Plant miRNAs are a class of endogenous non-coding small RNAs with 20–24 nt in length, which play essential roles in the regulation of gene expression at the transcription or post-transcription level [11–14]. MiRNAs regulate various biological processes, including organ development and hormone response [15–18]. Noticeably, an increasing number of studies have demonstrated that miRNAs play critical roles in plant drought tolerance and avoidance [19, 20]. Drought-tolerance related miRNAs and their targets have been identified in many plant species, including *Arabidopsis* [21–23], rice [24–26], maize [27], cotton [28], wheat [29] and soybean [30]. However, information is still very limited about miRNAs and their targets in tomato under drought stress. Only recently, Candar-Cakir et al. [9] have identified miRNAs and their targets in tomato seedlings treated with 5% polyethylene glycol to imitate drought stress.

Tomato (*Solanum lycopersicum*) is one of the most important vegetables grown globally [31]. However, cultivated tomato is sensitive to drought stress. Drought impairs nutrition uptake and root growth, and consequently reduces tomato yield and fruit quality [32–34]. Fortunately, wild tomato species possesses many useful genetic variations, especially the genes for biotic- and abiotic-stress resistance. For instance, wild tomato *S. pennellii* is a distant relative of *S. lycopersicum*, which inhabits the extremely dry region of the Peruvian Andes [35]. This desert species shows a series of unusual morphology, such as small green-fruits, sticky and thick leaves, and thick hairs [35, 36]. Using the drought-tolerant *S. pennellii* LA0716 as the donor and the drought-sensitive processing tomato cultivar M82 as the current patent, Eshed and Zamir [37] have constructed an introgression line population, which covers the whole genome of LA0716. Each introgression line contains only one chromosomal fragment from LA0716, thus any phenotypic variation can be traced back to the introgressed fragment [38]. It has been proved to be an ideal population in the dissection of complex QTL traits in tomato [36]. The genomes of LA0716 and M82 have been decoded, together with other genome data from sequencing and resequencing projects, which will accelerate the identification of genetic variation in tomato drought tolerance [35, 38, 39].

Considerable efforts have been undertaken in tomato to identify drought-tolerant QTLs or genes. Foolad et al. [40] have identified four drought-tolerant QTLs regarding seed germination. Gur and Zamir [41] have identified three QTLs using the introgression population of LA0716 (IL7–5–5, IL8–3 and IL9–2–5), which contribute to yield under drought condition. Gong et al. [42] have utilized repeated drought stress treatment and characterized two drought-tolerant introgression lines (IL2–5 and IL9–1) from the above introgression population. Besides, genome-wide association analysis has been applied to identify genetic variation on fruit quality components upon drought stress [43]. Transcriptome analysis based on microarray or sequencing was employed to identify drought-responsive genes in tomato [9, 33, 42]. A few of them have been further verified by transgenic approach, such as DREB, TAS14, USP and miRNA169 [44–47].

Despite that drought-stress related miRNAs have been documented in many plant species, information regarding miRNAs and their targets in tomato under drought stress condition is rare. Here, we characterized both miRNAs and mRNAs in the drought-tolerant introgression line, IL9–1, and its recurrent parent, M82. Dozens of miRNAs and thousands of protein-coding genes were identified to be differentially expressed upon drought stress in the two tomato genotypes tested. Gene Ontology and pathway analysis revealed significant changes in the pathways of hormone signal transduction, oxidative phosphorylation, phosphatidylinositol and peroxisome.

## Results

### Verification of drought tolerance of the two tomato genotypes

**Fig. 1:**
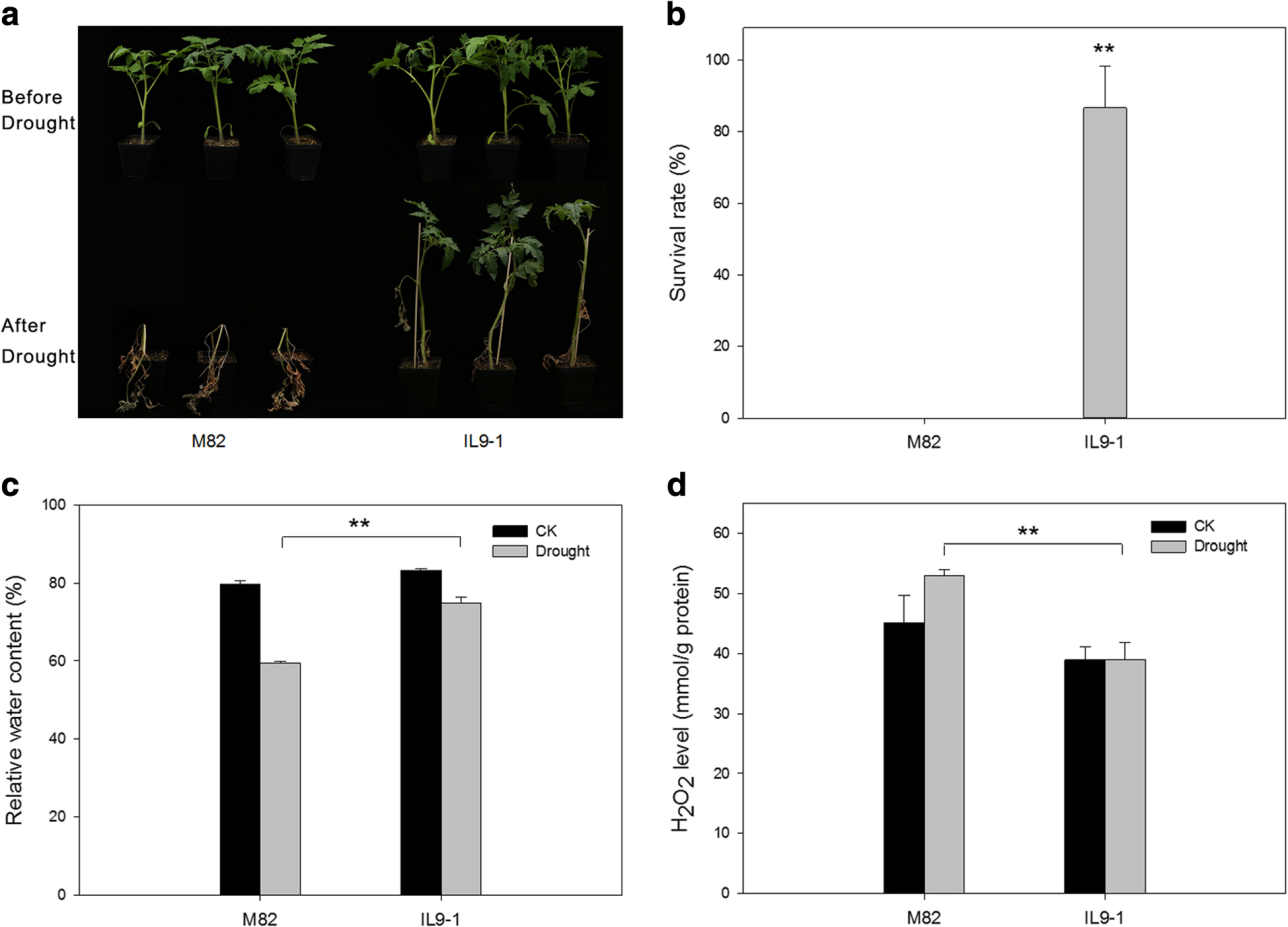
Drought tolerance of M82 and IL9–1. M82, a drought-sensitive genotype; IL9–1, a drought-tolerant introgression line with M82 as genetic background. **a** Phenotype of the seedlings after three weeks of drought stress and followed by a recovery. **b** Survival rate of the seedlings after the recovery. **c** Relative water content in leaf tissue ten days after drought stress. **d** Endogenous H_2_O_2_ content in leaf tissue ten days after drought stress. ** indicate a significant difference at *P* < 0.01 using two-tailed Students t-test

In a previous study, M82 and IL9–1 were identified as drought-sensitive and -tolerant genotype, respectively [42]. To verify this, seedlings of IL9–1 and M82 were challenged with drought and followed by a recovery. After recovery, the seeding survival rate of IL9–1 was about 87%, while it was zero for M82 (Fig. 1a, b). Ten days post the drought treatment, the leaf relative water content of IL9–1 (74.8%) was significantly higher than that of M82 (59.4%) (Fig. 1c). In addition, the H_2_O_2_ content in M82 was increased after drought treatment, while its level in IL9–1 remained unchanged, which resulted in a significant difference on H_2_O_2_ content between the two genotypes post drought stress (Fig. 1d). All these data confirmed that IL9–1 performed superior than M82 under drought stress.

### Sequencing results of small RNA libraries

**Fig. 2:**
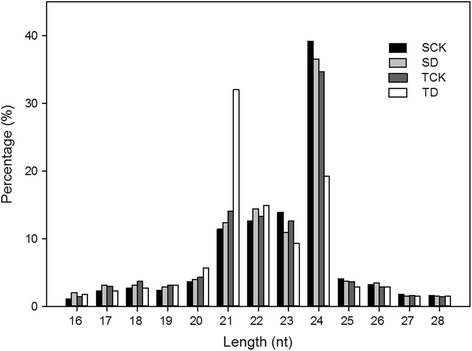
Length distribution of small RNAs in the four libraries from tomato. SCK: miRNA library from the drought-sensitive genotype M82 without drought stress, SD: from M82 with drought stress, TCK: from the drought-tolerant genotype IL9–1 without drought stress, TD: from IL9–1 with drought stress

To identify drought-stress related miRNAs in tomato, four small RNA libraries were constructed and sequenced for the RNA samples from the drought-tolerant introgression line IL9–1 without (TCK) and with (TD) drought stress, and also the drought-sensitive recurrent parent M82 without (SCK) and with (SD) drought stress. About 11–15 million raw reads were generated for each library (Table 1). Clean reads were then obtained by removing adaptors, junk and low-quality reads. Then, small RNAs within 16–28 nt were submitted to further analysis. The lengths of small RNAs were mainly distributed between 21 and 24 nt, which account for an average of 75.5% of the small RNAs (Fig. 2). However, there was a slight difference for the length distribution among different small RNA libraries. In the library of TD, small RNAs with 21 nt displayed the highest percentage (32.1%), while in the other three libraries, 24 nt small RNAs had the highest ratio (36.8% in average) (Fig. 2).

**Table 1:** Summary of small RNA sequencing data in the four libraries from tomato

Type	SCK		SD		TCK		TD	
	Count	Percentage (%)	Count	Percentage (%)	Count	Percentage (%)	Count	Percentage (%)
Raw reads	15,183,983	100	11,516,443	100	11,437,462	100	11,301,325	100
clean reads	14,650,320	96.49	11,120,683	96.56	11,203,392	97.95	11,109,195	98.30
Mapped	8,234,412	54.23	6,222,405	54.03	7,056,971	61.70	5,772,844	51.08
rRNA	98,876	0.65	130,051	1.13	109,746	0.96	70,194	0.62
tRNA	6958	0.05	7050	0.06	7037	0.06	6223	0.06
snRNA	31,275	0.21	25,056	0.22	29,932	0.26	38,310	0.34
snoRNA	26,290	0.17	50,073	0.43	25,608	0.23	53,981	0.48
miRNA	393,896	2.59	344,788	2.99	400,662	3.50	447,865	3.96
other ncRNA	336,947	2.22	221,085	1.92	362,003	1.66	262,282	2.32

### Conserved miRNAs in tomato under drought stress

To identify conserved (known) miRNAs in tomato, all the clean reads were blasted against the tomato known miRNAs in miRBase 21.0 database. A total of 105 conserved miRNAs were identified, which belong to 46 miRNA families (Additional file 1: Table S1). The numbers of miRNAs varied in different miRNA families, with the most members (seven) in sly-miR156 and sly-miR482, followed with five miRNAs in the family of sly-miR171. Noticeably, none of the conserved miRNAs was found located in the region corresponding to the introgressed chromosome segment of IL9–1.

**Fig. 3:**
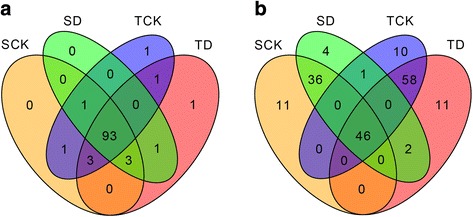
Venn diagram of tomato miRNAs identified in the four libraries. **a** conserved miRNAs. **b** novel miRNAs. SCK: miRNA library from the drought-sensitive genotype M82 without drought stress, SD: from M82 with drought stress, TCK: from the drought-tolerant genotype IL9–1 without drought stress, TD: from IL9–1 with drought stress

**Fig. 4:**
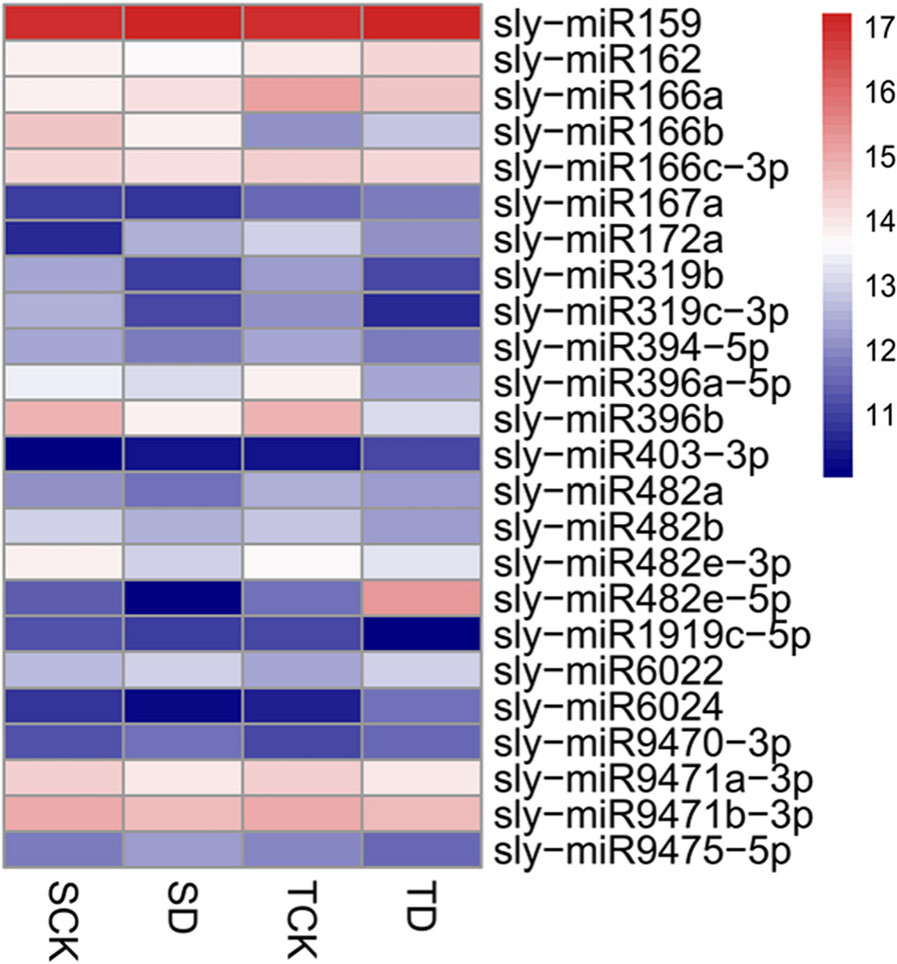
The most-abundant conserved miRNAs in the four libraries (reads number ≥ 1000; log2 transformed). SCK: miRNA library from the drought-sensitive genotype M82 without drought stress, SD: from M82 with drought stress, TCK: from the drought-tolerant genotype IL9–1 without drought stress, TD: from IL9–1 with drought stress. *Color scale* indicating log2 (reads)

The distribution of the 105 conserved miRNAs among different libraries was further investigated. Of them, 93 miRNAs were identified in all the four libraries (Fig. 3a). No miRNA was found to be specifically expressed in the drought-sensitive genotype M82, regardless of drought treatment. However, three miRNAs were expressed specifically in the drought-tolerant introgression line, in which sly-miR9469-3p was detected in TCK, sly-miR9472-3p in TD, and sly-miR164b-3p in both libraries (Fig. 3a, Additional file 1: Table S1). The expression level of different miRNAs varied in a wide range, with reads number from only a few to hundreds of thousands. There were 24 miRNAs with relatively higher expression level, with over 1000 reads across all the four libraries. These miRNAs belong to 16 families (Fig. 4, Additional file 1: Table S1).

**Fig. 5:**
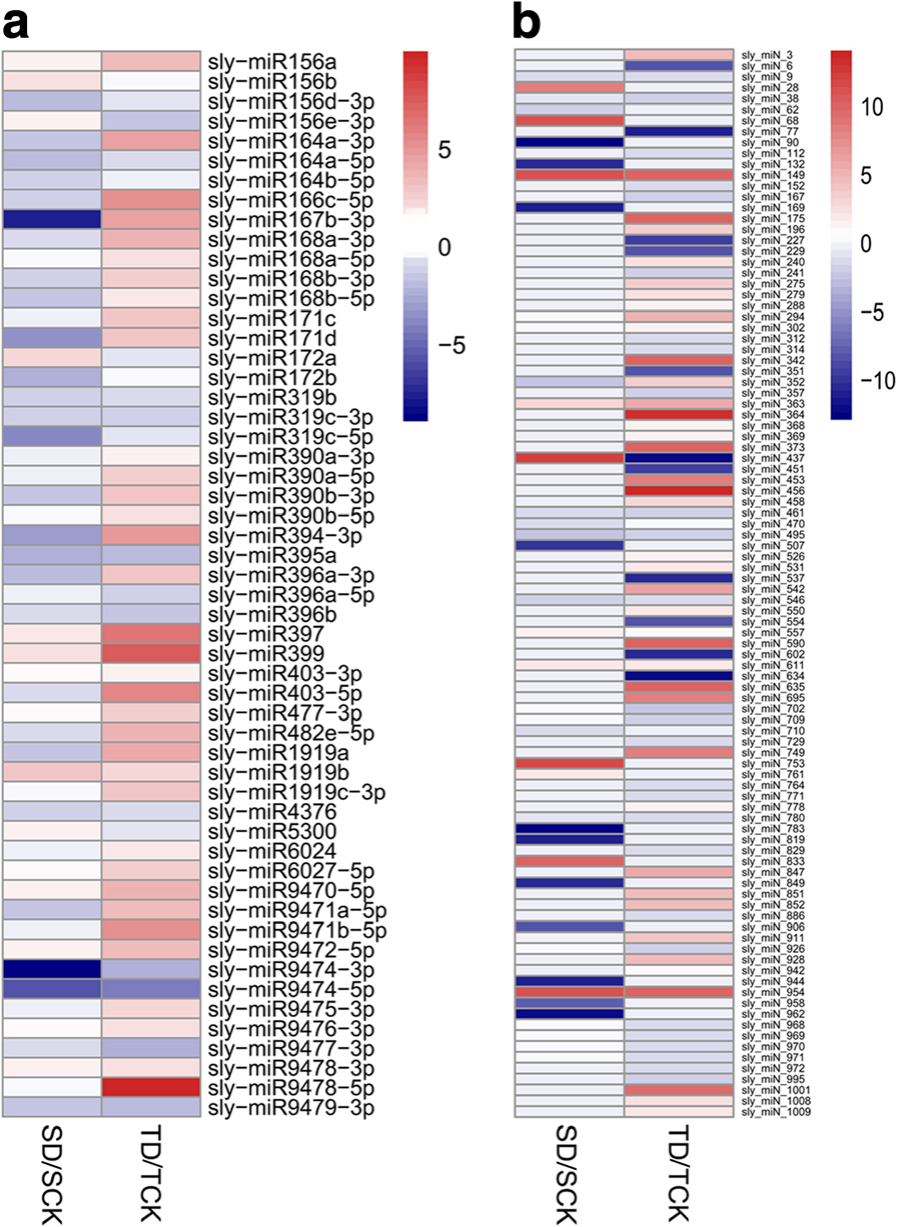
Differentially-expressed miRNAs upon stress treatment in drought-tolerant IL9–1 (T) and the sensitive genotype M82 (S). **a** conserved miRNAs. **b** novel miRNAs. D: drought treatment, CK: non-stress condition. *Color scale* indicating log2 (fold-change)

Under drought stress, 54 of the 105 conserved miRNAs (belonging to 31 families) were expressed differentially (|log2 fold-change| ≥ 1 and q-value ≤0.05) (Fig. 5a, Additional file 1: Table S1). In the drought-sensitive genotype M82, 24 conserved miRNAs were found to be differentially expressed after stress. Among them, 5 were up-regulated and 19 were down-regulated (Additional file 1: Table S1). While in the drought-tolerant genotype IL9–1, a total of 43 conserved miRNAs were differentially expressed, with 35 up-regulated and 8 down-regulated (Additional file 1: Table S1). The two genotypes shared 13 differentially-expressed conserved miRNAs, but only four of them had similar expression patterns, in which sly-miR156a and sly-miR1919b were up-regulated while sly-miR9474-3p and sly-miR9474-5p down-regulated. Nine miRNAs (sly-miR164a-3p, sly-miR166c-5p, sly-miR167b-3p, sly-miR168b-3p, sly-miR168b-5p, sly-miR394-3p, sly-miR396a-3p, sly-miR1919a and sly-miR9471a-5p) were down-regulated in M82, whereas they were up-regulated in IL9–1 (Fig. 5a). In addition, 11 and 30 differentially-expressed miRNAs were detected to be specifically expressed in M82 and IL9–1, respectively (Fig. 5a, Additional file 1: Table S1).

### Novel miRNAs in tomato under drought stress

To identify putative novel miRNAs in tomato, novel miRNAs were predicted using miR-PREFeR. A total of 179 novel miRNAs were predicted, among them, 46 were detected in all the four small RNA libraries (Fig. 3b, Additional file 2: Table S2). Different numbers of novel miRNAs were detected among libraries. For the drought-sensitive genotype, 93 and 89 novel miRNAs were detected in the library of SCK and SD, respectively, and 82 of them were common (Fig. 3b). For the drought-tolerant genotype, 115 and 117 were detected in TCK and TD, respectively, and 104 were common (Fig. 3b). Two novel miRNAs (sly_miN_149 and sly_miN_954) were described as drought-specific miRNA, which were detected only in the drought-stressed samples (Additional file 2: Table S2). Some novel miRNAs were described as genotype-specific miRNA, for instance, sly_miN_62 and sly_miN_710 were detected only in M82, while sly_miN_112 and sly_miN_386 were detected only in IL9–1 (Additional file 2: Table S2). As expected, most novel miRNAs had a relatively low expression, and few of them had reads over 1000. For instance, sly_miN_294 had 15,199 reads in the miRNA library TD, while sly_miN_945 had 1101 reads in the library TCK (Additional file 2: Table S2).

Differential expression analysis was also applied on the novel miRNAs. 98 of the 179 novel miRNAs were differentially expressed (|log2 fold-change| ≥ 1 and q-value ≤0.05) under drought stress treatment (Fig. 5b, Additional file 2: Table S2). In the drought-sensitive genotype M82, 30 novel miRNAs were found to be differentially expressed after stress, of them, 11 were up-regulated and 19 were down-regulated. While in the drought-tolerant genotype IL9–1, a total of 78 novel miRNAs were differentially expressed, with 40 up-regulated and 38 down-regulated. The two genotypes shared 10 differentially-expressed novel miRNAs, and four of them (sly_miN_149, sly_miN_363, sly_miN_611 and sly_miN_954) were up-regulated and also four of them (sly_miN_9, sly_miN_461, sly_miN_495 and sly_miN_546) were down-regulated (Additional file 2: Table S2). The other two miRNAs had different expression patterns in the two genotypes under stress. Sly_miN_352 was down-regulated in M82 while it was induced in IL9–1. A reverse pattern was detected for sly_miN_437, which was induced in M82 but down-regulated in IL9–1 (Additional file 2: Table S2).

Noticeably, genes encoding four of the novel miRNAs (sly_miN_702, sly_miN_707, sly_miN_709 and sly_miN_710) were found to be located in the chromosome region corresponding to the introgressed region of IL9–1. Of them, three were differentially expressed under drought stress: sly_miN_702 and sly_miN_709 were down-regulated in IL9–1 and sly_miN_710 was down-regulated in M82.

### Differential expressed genes and pathways in tomato under drought stress

To investigate drought responsive genes as well as target genes of the miRNAs in tomato, four RNA samples in duplicate were subjected for sequencing, including samples from the drought-tolerant IL9–1 without (TCK) and with (TD) drought stress, and the drought-sensitive M82 without (SCK) and with (SD) stress. A total of 336,961,324 raw reads and 322,139,020 clean reads were obtained (Table 2). Among the clean reads, 297,005,379 could be mapped to the tomato reference genome (https://solgenomics.net, version SL2.50), which accounted for 92.20% of the total cleans reads. Of them, 286,705,237 were unique mapped reads and the rest were multiple mapped reads (Table 2).

**Table 2:** Statistics of the reads alignments in the RNA-Seq study

Sample	Raw reads	Clean reads	Total mapped reads	Unique mapped reads	Multiple mapped reads	Mapping percentage (%)
SCK-1	41,554,238	39,494,120	35,701,154	34,246,056	1,455,098	85.91
SCK-2	43,797,840	42,091,578	38,448,660	37,198,941	1,249,719	87.79
SD-1	35,869,978	34,450,564	32,184,967	31,189,526	995,441	89.73
SD-2	46,885,350	45,052,578	42,435,372	41,163,197	1,272,175	90.51
TCK-1	42,080,970	40,148,064	36,969,596	35,403,809	1,565,787	87.85
TCK-2	42,751,392	40,515,224	37,020,007	35,729,643	1,290,364	86.59
TD-1	41,450,786	39,775,940	36,783,188	35,486,170	1,297,018	88.74
TD-2	42,570,770	40,610,952	37,462,435	36,287,895	1,174,540	88.00
Average	42,120,165.5	40,267,377.5	37,125,672.4	35,838,154.6	1,287,517.8	88.14

**Fig. 6:**
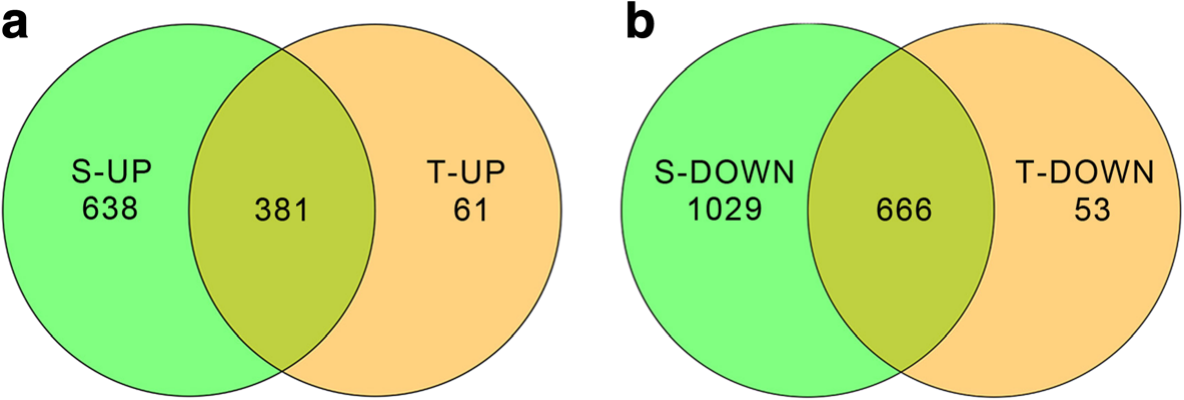
Venn diagram showing the numbers of up- and down-regulated genes upon stress treatment. T: Drought-tolerant genotype IL9–1, S: Drought-sensitive genotype M82. **a** Up-regulated (UP) differentially-expressed genes. **b** Down-regulated (DOWN) differentially-expressed genes

After gene annotation and expression analysis, a total of 18,085 genes were detected in the RNA samples of M82. Among them, 2714 were expressed differentially (|log2 fold-change| ≥ 1 and q-value ≤0.05), with 1019 up-regulated and 1695 down-regulated (Additional file 3: Table S3). While in IL9–1, the drought-tolerant genotype, 17,289 genes were detected. Among them, only 1161 were differentially expressed, with 442 up-regulated and 719 down-regulated (Additional file 4: Table S4). There were 381 up-regulated genes shared by the two genotypes, and the numbers of genes induced specifically in M82 and IL9–1 were 638 and 61, respectively (Fig. 6a). There were 666 common down-regulated genes in the two genotypes tested, and 1029 and 53 down-regulated genes specific for M82 and IL9–1, respectively (Fig. 6b).

**Fig. 7:**
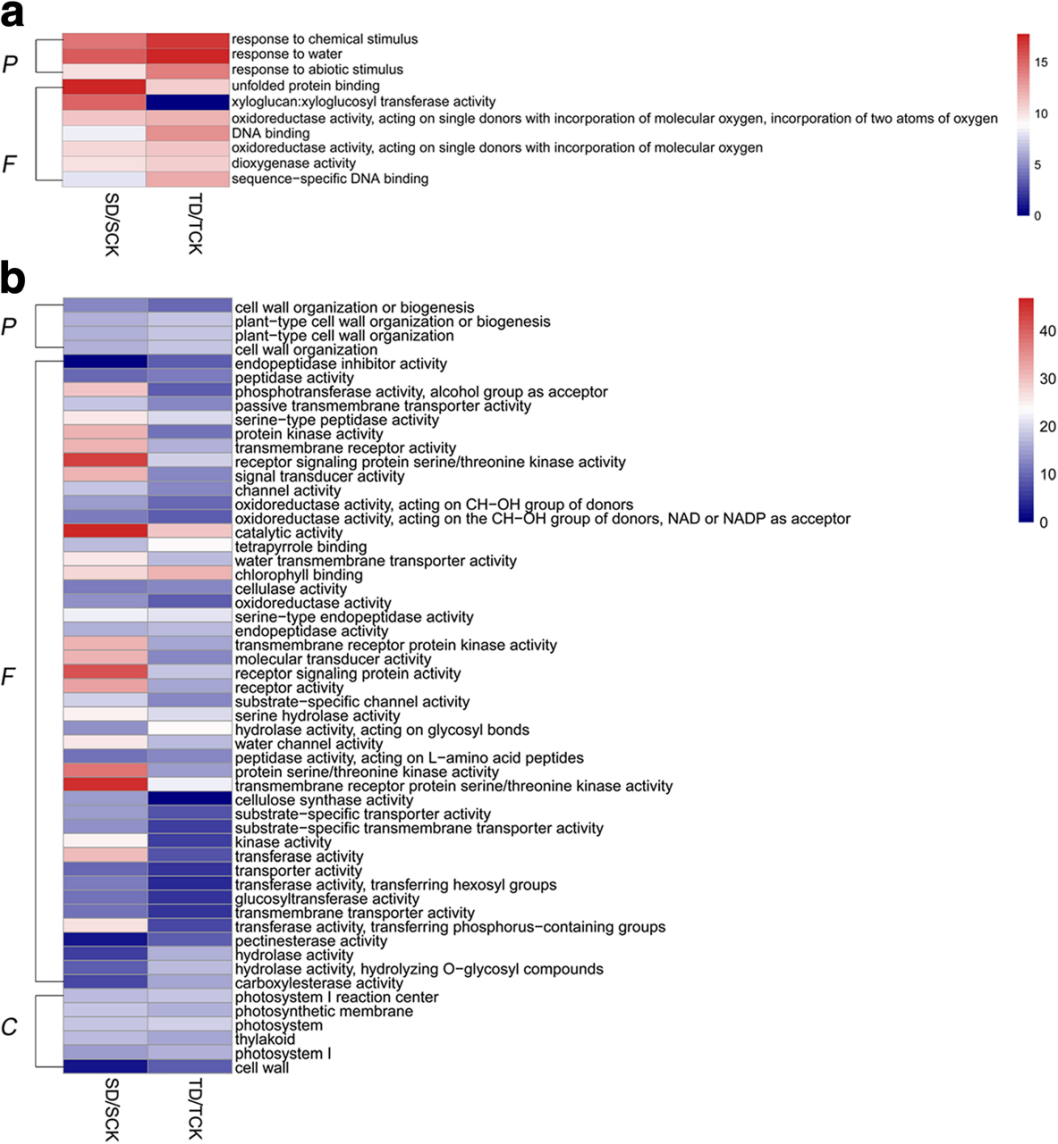
The *p*-value (-log2 transformed) heatmap of significant GO terms under drought condition in tomato. **a** Significantly-enriched GO terms for the up-regulated differentially-expressed genes. **b** Significantly-enriched GO terms for the down-regulated differentially-expressed genes. P: Biological Process. F: Molecular Function. C: Cellular Component. *Color scale* indicating -log2 (*p*-value)

Further, gene ontology (GO) enrichment and pathway analysis based on KEGG (Kyoto Encyclopedia of Genes and Genomes) database were applied on the differentially-expressed genes under drought treatment. Regarding the up-regulated genes under drought stress, four and nine significantly enriched GO terms were identified for M82 and IL9–1, respectively (Fig. 7a, Additional file 5: Table S5a, b). The biological process GO term “response to water” was significantly enriched both in M82 and IL9–1, while the GO term “response to abiotic stimulus” was only significantly enriched in IL9–1, the drought-tolerant genotype (Fig. 7a). As for the down-regulated genes under drought stress, 49 and 45 significant GO terms were identified for M82 and IL9–1, respectively (Fig. 7a; Additional file 5: Table S5c, d). Four biological process GO terms were significantly enriched both in M82 and IL9–1, which were related to cell wall biogenesis and organization (Fig. 7b). Regarding to the down-regulated genes, many molecular function GO terms related to transferase and kinase activity were significantly enriched, especially for the drought-sensitive genotype (Fig. 7b). The cellular component GO terms related to photosynthesis were enriched after drought-stress treatment.

Pathway analysis based on KEGG database was carried out. For the 1019 up-regulated genes in the drought-sensitive genotype M82, a total of 95 pathways involving 245 genes were retrieved (Additional file 6: Table S6). For the 442 up-regulated genes for IL9–1, 74 pathways were retrieved, which involved 118 genes (Additional file 7: Table S7). For the down-regulated genes, 109 (418 out of the 1695 genes) and 84 pathways (189 out of the 719 genes) were identified for M82 and IL9–1, respectively (Additional file 8: Table S8 and Additional file 9: Table S9). As expected, many pathways related to plant stress tolerance have been retrieved, such as pathways related to plant hormone signal transduction, oxidative phosphorylation, phosphatidylinositol signaling system and peroxisome. Among them, the number of pathway involved in plant hormone signal transduction was higher, which was only less than that related to metabolism. After drought treatment, 46 and 22 genes involved in plant hormone signal transduction were differentially expressed in M82 and IL9–1, respectively.

Regarding to the introgression region corresponding to IL9–1, 53 and 11 differentially-expressed genes were identified in the drought-sensitive (M82) and -tolerant (IL9–1) genotype, respectively. Among them, eight genes were identified in both genotypes, they were Solyc09g005060, Solyc09g007270, Solyc09g007750, Solyc09g008280, Solyc09g008860, Solyc09g009770, Solyc09g010210 and Solyc09g010860. Of these eight genes, Solyc09g007270, Solyc09g007750, Solyc09g008860, Solyc09g009770 and Solyc09g010860 were related to abiotic-stress tolerance, which encode ascorbate peroxidase, two receptor kinases, calmodulin-binding protein and expansin. Three differentially expressed genes (Solyc09g005690, Solyc09g008990 and Solyc09g009010) were specifically detected in IL9–1 in this region, however, their expression levels were low.

### Target gene prediction and functional classification of the drought-induced conserved miRNAs in tomato

The target genes of all the 54 conserved miRNAs which were differentially expressed upon drought stress were predicted using the psRNATarget server. A total of 274 targets were found for 47 of the 54 miRNAs (Additional file 10: Table S10), the rest seven miRNAs (sly-miR156e-3p, sly-miR394-3p, sly-miR403-5p, sly-miR1919a, sly-miR1919b, sly-miR1919c-3p and sly-miR9471b-5p) had no targets. The predicted target genes mainly encode transcription factors, drought-stress related proteins, pathogenesis-related proteins, kinase, phosphatase, proteinase in signal transduction pathway, and enzymes in various metabolisms. For instance, the target of sly-miR156a, sly-miR164a-5p, sly-miR171c, sly-miR171d and sly-miR319c-3p were genes encoding SPL, NAC, MYB, GRAS and TCP transcription factors, respectively, which are all related to plant stress tolerance (Additional file 10: Table S10). Some targets of miRNAs have been documented to be involved in plant disease resistance, such as the targets of sly-miR477-3p, sly-miR5300 and sly-miR6024 (Additional file 10: Table S10). Some target genes of the conserved miRNAs encode drought-stress related proteinase, such as the targets of sly-miR396 and sly-miR397, which encode cysteine proteinase and laccase, respectively. Some targets encode protein kinase and phosphokinase, such as the targets of sly-miR156a, sly-miR390a-5p, sly-miR482e-5p and sly-miR6027–5p. The targets of sly-miR172a, sly-miR390b-5p, and sly-miR6024 encode AP2-like ethylene-responsive transcription factor, protein phosphatase 2C, and auxin-independent growth promoter protein-like protein, respectively; these genes function in hormone signal transduction pathways (Additional file 10: Table S10).

**Fig. 8:**
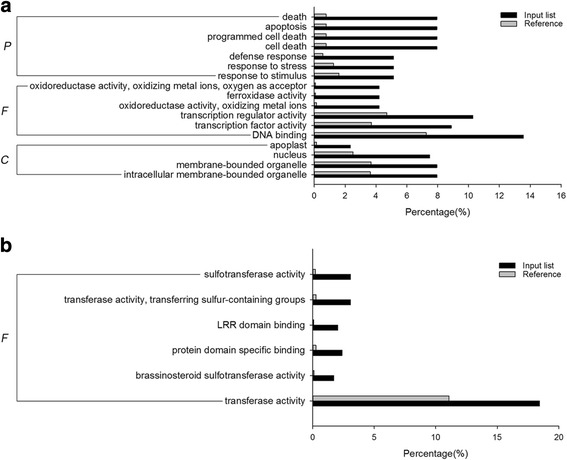
Significantly enriched GO terms for the target genes of conserved (**a**) and novel (**b**) miRNAs. The percentage for the input list is calculated by the number of genes mapped to the GO term divided by the number of all genes in the input list. The same calculation was applied to the reference list to generate its percentage. P: Biological Process, F: Molecular Function, C: Cellular Component

The 274 target genes of the differentially-expressed conserved miRNAs were further submitted to GO and KEGG pathway analysis. It was found that the target genes were classified into 18, 14 and 38 GO terms from the biological process, cellular component and molecular function ontology, respectively. Among them, 17 significantly enriched GO terms were identified (Additional file 11: Table S11). GO analysis showed that the target genes of many differentially-expressed miRNAs were related to plant drought stress tolerance, specially the target genes matching GO terms that were significantly enriched (Fig. 8a, Additional file 11: Table S11). Enrichment of biological process GO terms was detected to be related to stress tolerance, such as defense response, response to stress and response to stimulus. Significantly enriched molecular function GO terms were found to be related to stress tolerance, such as oxidoreductase activity, oxidizing metal ions, oxygen as acceptor, and transcription factor activity (Fig. 8a). The 274 genes were also submitted to KEGG pathway analysis, 88 of them were mapped to 45 pathways (Additional file 12: Table S12). The highest number of target genes were detected in metabolic pathways (16 genes), followed by biosynthesis of secondary metabolites (5 genes). The number of target genes in other pathways varied from one to four. Pathways such as phosphatidylinositol signaling system, peroxisome and plant hormone signal transduction were supposed to be involved in plant stress tolerance.

The expression of the 274 miRNA target genes was further analyzed using the RNA-Seq data, and 195 genes were detected in the RNA-Seq datasets that targeted by the differentially expressed miRNAs (Additional file 13: Table S13). The changing pattern of most targets was in line with the expectation, nevertheless, the expression level of only a small portion of the target genes changed significantly.

### Target gene prediction and functional classification of the drought-induced novel miRNAs

Similar to conserved miRNAs, target genes were also predicted for the 98 differentially-expressed novel miRNAs. A total of 410 target genes were predicted, with no target for nine novel miRNAs (sly_miN_77, sly_miN_149, sly_miN_175, sly_miN_453, sly_miN_470, sly_miN_495, sly_miN_635, sly_miN_778 and sly_miN_954). The target genes of the novel miRNAs were mainly involved in cellular process, metabolism process and catalytic activities (Additional file 14: Table S14). As expected, some target genes of the novel miRNAs were also involved in plant stress tolerance. For instance, the target genes of sly_miN_368, sly_miN_456 and sly_miN_531 encode WRKY, bZIP and MYB transcription factor, respectively. The targets of sly_miN_169, sly_miN_314, sly_miN_847 encode receptor-like protein kinases, a target of sly_miN_279 encodes serine-threonine protein kinase, and a target of sly_miN_279 encodes LRR receptor-like serine/threonine-protein kinase. These genes were highly related to plant stress tolerance (Additional file 14: Table S14).

As like conserved miRNAs, GO and KEGG pathway analyses were applied on the target genes of the novel miNRAs. Six significantly enriched molecular function GO terms were obtained, including GO terms related to sulfotransferase activity, transferring sulfur-containing groups, LRR domain binding, protein domain specific binding, brassinosteroid sulfotransferase activity and transferase activity (Fig. 8b, Additional file 15: Table S15). Among them, GO terms involved in LRR domain binding and transferase activity were related to plant stress tolerance. In addition, a total of 75 target genes could be mapped to 80 pathways (Additional file 16: Table S16). It was shown that the highest numbers of genes were detected in metabolic pathways and biosynthesis of secondary metabolites. Many of the pathways are related to stress tolerance, such as pathways of peroxisome and plant hormone signal transduction.

Based on the RNA-Seq results, transcripts were detected for 280 target genes of the novel miRNAs (Additional file 17: Table S17).

### Network of drought-responsive miRNA and their targets

**Fig. 9:**
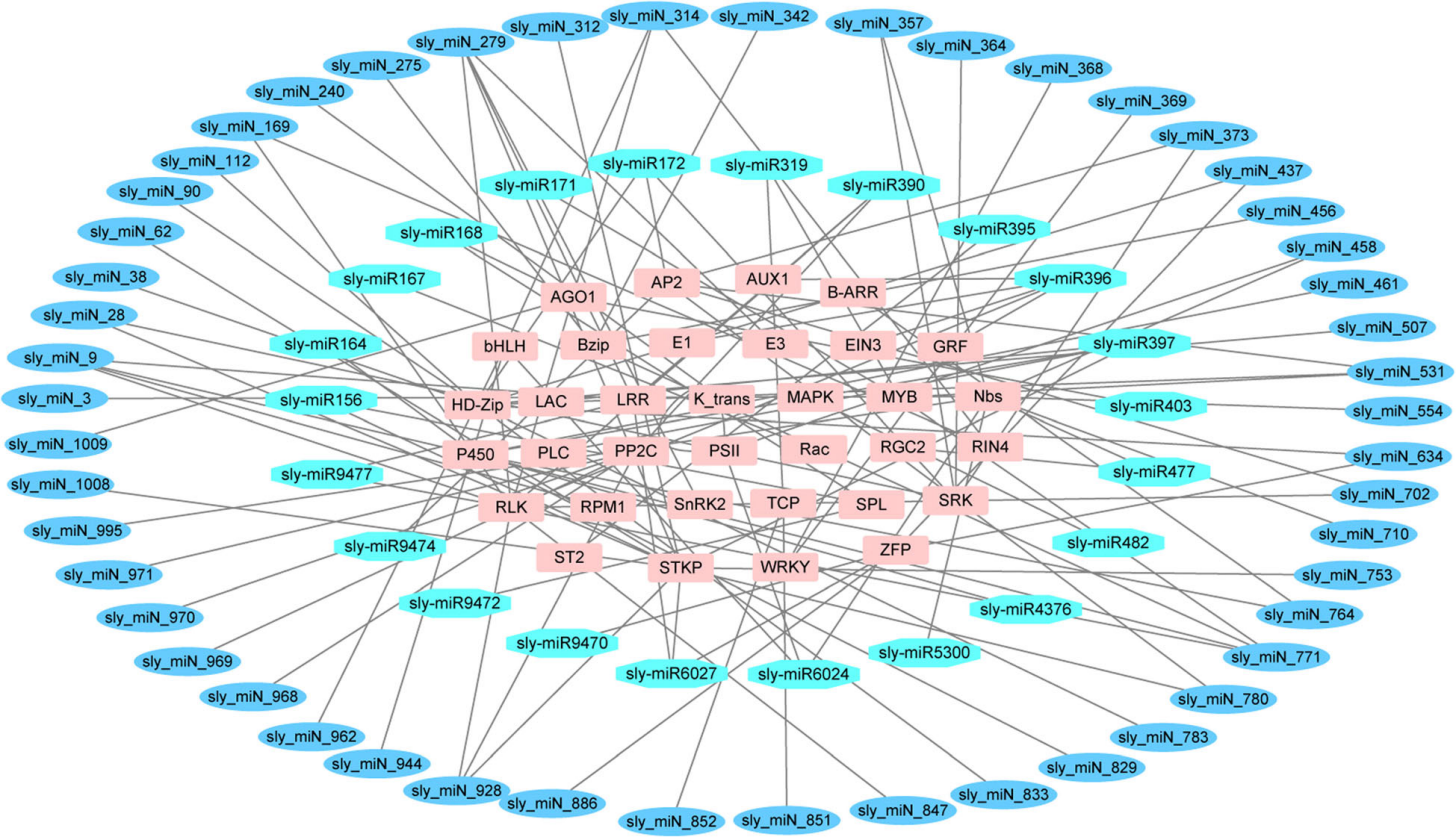
Network analysis between miRNA and their potential drought-responsive targets. Network analysis was performed using the Cytoscape network platform

To investigate the relationship between drought-responsive miRNA and their targets, network analysis was carried out using the Cytoscape platform. The analysis incorporated 72 miRNAs including 27 conserved miRNAs belonging to 22 families and 50 novel miRNAs, together with 34 genes involved in stress tolerance or plant development, such as genes encoding transcription factors, protein kinases and phosphatases, and hormone-responsive factors (Fig. 9). It was found that different miRNA targeted different number of stress-related genes. For instance, the conserved miRNA sly-miR9472 targeted only one gene (SPL), while sly-miR396 targeted five genes (AUX1, E1, EIN3, GRF and RLK). Similarly, the novel miRNA sly_miN_373 targeted two genes (AP2 and SRK), but sly_miN_279 targeted five genes (E3, LRR, RLK, SnRK2 and STKP).

### qRT-PCR validation of miRNA and mRNA expression

**Fig. 10:**
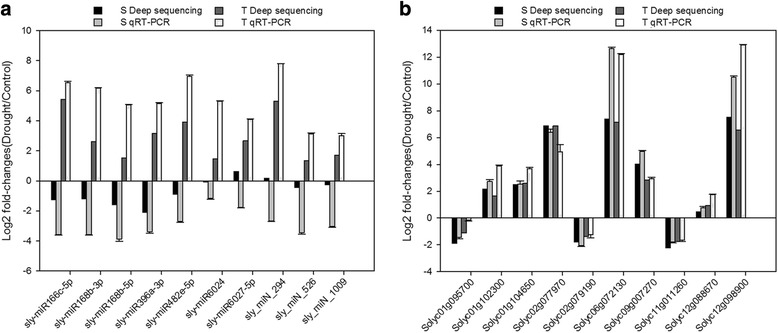
qRT-PCR validation of drought-responsive miRNAs (**a**) and genes (**b**) in tomato. The 2^-ΔΔCt^ was used to calculate the fold change of expression in qRT-PCR analysis, with U6 and Actin 4 as reference for miRNA and genes respectively. All experiments were repeated three times, and the expression data were log2 transformed before analysis. Error bars represent the standard deviation of the replicates. The deep sequencing results with log2 fold change are shown here to compare with qRT-PCR results. S: a drought-sensitive cultivar, M82, T: a drought-tolerant introgression line, IL9–1

Quantitative RT-PCR was employed to verify the miRNA/gene expression results. Seven conserved miRNAs (sly-miR166c-5p, sly-miR168b-3p, sly-miR168b-5p, sly-miR396a-3p, sly- miR482e-5p, sly-miR6024 and sly-miR6027-5p) and three novel miRNAs (sly_miN_294, sly_miN_526 and sly_miN_1009) were selected in the validation. In the drought-sensitive genotype, M82, the results of the miRNAs from sequencing were in accordance with those from qRT-PCR analysis, except for sly-miR6027-5p and sly_miN_294, which was up-regulated in the small RNA sequencing but down-regulated in qRT-PCR analysis (Fig. 10a). In the drought-tolerant IL9–1, all the results from small RNA sequencing were in line with the results from qRT-PCR (Fig. 10a). Ten genes were also selected to validate the RNA-sequencing results, including three genes in hormone-response pathway (Solyc01g095700, Solyc02g079190 and Solyc11g011260), five genes in drought responsiveness (Solyc02g077970, Solyc06g072130, Solyc09g007270, Solyc12g088670 and Solyc12g098900), and two genes encoding transcription factors (Solyc01g102300, Solyc01g104650). Consistent results were obtained from RNA sequencing and qRT-PCR, suggesting that the quality was high for the RNA-Seq datasets (Fig. 10b).

## Discussion

Numerous evidences have demonstrated that “omic” technologies have become one of the most powerful tool in system biology study, which could help efficiently decipher the mechanisms underlying plant stress tolerance [8]. Under drought stress, plant mobilizes many defense mechanisms to cope with the environmental challenge, which involves molecular, biochemical and physiological changes [48]. In this research, we employ high-throughput sequencing of miRNA and mRNA to address the molecular differences between two tomato lines with distinct performance on drought tolerance.

### Drought-responsive characteristics of M82 and IL9–1

Tomato is one of the most important vegetable species grown worldwide. However, tomato yield and fruit quality were adversely affected by drought stress [31, 33]. Unlike cultivated tomato, elite genes on drought tolerance were enriched in wild tomato species. However, due to the complexity of trait and the distinct genetic background, it remains challenging to genetically dissect the quantitative trait variation of drought tolerance in wild tomato species [36, 49]. Fortunately, Eshed and Zamir [37] have constructed an introgression line population covering the whole genome of LA0716, an extremely drought-tolerant wild tomato accession. An introgression line carries only one chromosomal fragment from the wild species, thus it can largely eliminate epistatic effects from different QTLs [50]. Gong et al. [42] have screened the introgression population of LA0716 and identified IL2–5 and IL9–1 as highly drought-tolerant lines. Here, we confirmed IL9–1 as a drought-tolerant line. After the recovery from a three-week treatment of drought stress, the survival rate of IL9–1 is high (87%) while all the seedlings of M82 died (Fig. 1a, b). After stress, the leaf relative water content was significantly higher in IL9–1 than in M82 (Fig. 1c). In addition, the H_2_O_2_ content in IL9–1 was significantly lower than that in M82 after stress, indicating that IL9–1 exhibited a higher capability to scavenge reactive oxygen species produced under drought stress. All these results suggested that the introgressed chromosomal region of IL9–1 carried drought-tolerant locus or loci.

### Conserved and novel miRNAs and their differential expression in tomato

MiRNA is a class of non-coding RNAs with 20–24 nt in length, which regulates gene expression at transcriptional and posttranscriptional level in plant and animal [15]. Increasing evidence demonstrated that miRNAs play important roles in plant response to abiotic stress [19, 20]. To date, drought-stress related miRNAs have been identified in many plant species through microarray or deep sequencing technologies [5, 19]. However, little work has been carried out to characterize drought tolerant miRNA. In this study, we employed deep sequencing technologies to investigate the miRNA and gene expression profile upon drought stress in two tomato genotypes (M82, a drought-sensitive cultivar, and IL9–1, a drought-tolerant introgression line with M82 as genetic background), which show distinct performance on drought tolerance [37, 42].

In the four small RNA libraries sequenced, a total of 105 conserved miRNAs belonging to 46 families and 179 novel miRNAs have been identified. Similar to previous reports from rice and tomato, different conserved miRNAs had different expression levels, with reads ranged from a few to hundreds of thousands [9, 24, 51]. As expected, most novel miRNAs had a low expression level, with reads less than 100, which also agreed with the previous results from tomato and *Arabidopsis* [51, 52].

Based on the transcriptome datasets, 54 conserved and 98 novel miRNAs were characterized as differentially expressed miRNAs. Similar to the results from tomato seedlings treated with polyethylene glycol [9], most of the differentially expressed miRNAs were down-regulated in the sensitive genotype, while the majority of them were up-regulated in the tolerant genotype. In our case, 19 out of the 24 differentially-expressed conserved miRNAs were down-regulated in M82, the drought-sensitive cultivar; however, 35 out of the 43 differentially-expressed conserved miRNAs were up-regulated in the drought-tolerant introgression line, IL9–1(Additional file 1: Table S1). In addition, we also detected a few miRNAs showing opposite pattern in M82 and IL9–1, this kind of results have also been observed in previous studies on rice, wheat and tomato [9, 24, 53]. Cases were sly-miR166c-5p, sly-miR169e-3p and sly-miR396a-3p, which were down-regulated in M82 but up-regulated in IL9–1. Many conserved miRNAs showed similar expression patterns across different plant species, suggesting their conserved function in particular biological process. For instance, the expression of miRNA156, miRNA169, miRNA172 and miRNA319 was significantly changed upon drought stress in tomato, and these miRNAs were also induced by drought stress in *Arabidopsis*, rice and wheat [9, 29, 54, 55]. Noticeably, some conserved miRNAs were firstly identified in our study to be drought-responsive in tomato, such as sly-miR1919, sly-miR5300 and sly-miR9477. Besides, we also found 98 of the 179 novel miRNAs changed their expression significantly upon drought stress. Among them, 20 and 68 were specific to M82 and IL9–1, respectively, and 10 were shared by the two genotypes but two of them (sly_miN_352 and sly_miN_437) showed opposite expression trend (Additional file 2: Table S2). Interestingly, no conserved miRNA was detected locating in the introgression region corresponding to IL9–1. However, four novel miRNAs (sly_miN_702, sly_miN_707, sly_miN_709 and sly_miN_710) were found to be in this region. Three of them (sly_miN_702, sly_miN_709, sly_miN_710) were differentially expressed under stress: sly_miN_702 and sly_miN_709 were expressed specifically in IL9–1, the drought-tolerant line, and sly_miN_710 was expressed specifically in M82, the sensitive cultivar. These novel miRNAs may contribute to the different performance of M82 and IL9–1 under drought stress condition.

Overall, we have identified a whole bunch of drought-responsive miRNAs. Twelve differentially-expressed miRNAs with similar expression trend were shared by the two genotypes (Additional file 1: Table S1 and Additional file 2: Table S2), indicating that they may play conserved roles in tomato drought response. However, more miRNAs showed different expression between the two genotypes, such as sly-miR396a-5p, sly-mi396b and sly_miN_780. This could be due to the introgression of chromosome 9 fragment from LA1706. This introgression might affect the expression of the above microRNAs directly or indirectly, thus improve the drought tolerance of IL9–1.

### RNA-seq and expression profiles of tomato under drought stress

Transcriptomic approach has been widely used to characterize expression profiles under various stress conditions in plants, and the quantification of mRNAs is generally based on microarray or RNA-Seq analysis [7, 8]. Microarray has been intensively used in the past decades, however, this technology has obvious limits. The number of genes profiled was limited to the probes printed in the slide, and probe synthesis has also to be based on known sequence. In the case of tomato, an oligo chip covers only one third of the tomato genes [42]. In addition, it would be difficult for microarray to detect low-abundant genes because of detective sensitivity [56, 57]. Comparably, RNA-Seq offers many benefits in transcriptome profiling, which can be used to detect gene expression unbiasedly, and it also enable us to detect novel transcripts and alternative splicing events [58, 59].

As an agronomically important vegetable species as well as a research model in plant science, tomato was also intensively studied regarding abiotic stress tolerance [35]. Transcriptomic tools have been widely used in studying stress responsiveness of tomato [42, 60, 61]. We utilized deep sequencing technology to compare the mRNA and miRNA expression between the drought-sensitive genotype M82 and the tolerant one IL9–1.

Under drought stress, 2714 and 1161 differentially-expressed genes were identified in M82 and IL9–1, respectively, which suggested that the responsiveness in the sensitive genotype was more obvious (Additional file 3: Table S3 and Additional file 4: Table S4). This feature has also been reported in a previous study based on microarray analysis [42]. GO and pathway analyses revealed that many differentially-expressed genes were involved in metabolism, physiological and biochemical processes, which is reasonable as plant has to adapt to environmental stress by adjusting basic cellular metabolism [42, 62]. As compared to the results of Gong et al. [42], besides the enrichment of biological process GO term of response to abiotic stimulus, additional GO terms related to stress were enriched in our study, such as response to water (e.g. genes encoding dehydrin and LEA protein), oxidation reduction and lipid transport [35, 46, 63]. Differentially-expressed genes were significantly enriched in GO term “response to water” both for IL9–1 and M82, indicating that these genes could be essential for the drought response of tomato. Noticeably, we found that the GO term “response to abiotic stimulus” was only significantly enriched in the drought tolerant line, IL9–1, suggesting that differentially-expressed genes in this GO biological category may contribute to the difference on drought tolerance between the two genotypes. Pathway analysis on differentially-expressed genes revealed various stress-related pathways, including pathways of plant hormone signal transduction, oxidative phosphorylation, phosphatidylinositol signaling system and peroxisome. Plant hormones are well-known to be critical in plant growth, development and stress tolerance [64, 65]. A total of 47 differentially-expressed genes were detected in plant hormone signal transduction pathways, indicating the essential involvement of hormones in the drought adaptation of tomato.

As expected, many differentially-expressed genes that closely involving drought tolerance, were found to be located in the introgression region of IL9–1. We noticed that a gene (Solyc09g007270) encoding ascorbate peroxidase is located in the introgressed region corresponding to IL9–1, and this gene was induced significantly by drought stress. Ascorbate has been well-documented to function positively in plant stress tolerance as it eliminates the reactive oxygen species produced by abiotic stresses [66]. Nucleotide and amino acid sequence variation was observed for this gene between the two genotypes (data not shown), and a previous report has shown that IL9–2–5, a sub-line of IL9–1, has a higher level of ascorbic acid than M82 [67]. According to our assessment, IL9–1 had a significantly lower level of H_2_O_2_ in leaf than M82 when the seedlings were challenged with drought stress (Fig. 1d). The difference on ascorbate peroxidase and ROS scavenging capability may partially explain the phenotypic difference between M82 and IL9–1 regarding drought tolerance.

### Target genes of miRNA in tomato

To study the potential role of miRNAs in tomato drought tolerance, their target genes were predicted and the gene expression was analyzed using the RNA-Seq datasets. Target genes were retrieved from 47 out of the 54 differentially-expressed conserved miRNAs, and it was found that many of them are related to plant stress tolerance, including genes encoding transcription factors, drought-tolerance related proteinase, pathogenesis-related proteins, protein kinase and phosphatase, proteinase in signal transduction pathways, and enzymes in various metabolic pathways. GO analysis enriched biological process GO terms of defense response, response to stress and response to stimulus.

Squamosa promoter binding protein-like (SPL) transcription factor genes are targets of sly-miR156a, and these genes are key regulators in plant vegetative-to-reproductive transition [68–70]. Overexpressing miRNA156 in *Arabidopsis* and rice results in reduced expression of SPL genes, and further affects the downstream genes *PAP1* and *DRF* that function in anthocyanin pathway. As a result, the transgenic plants show improved performance under drought and salt stresses [71]. In our study, the expression of sly-miR156a increased significantly in M82 and IL9–1, suggesting its conserved roles in development and stress tolerance. Further analysis showed 10 out of the 13 target genes of sly-miR156a were SPL genes, and four of them (Solyc03g114850, Solyc07g062980, Solyc10g009080 and Solyc10g078700) were down-regulated by drought stress both in M82 and IL9–1. Our results indicated that sly-miR156a and its target genes appear to play a conserved role in tomato drought response.

It was found that after drought stress, sly-miR396a-5p and sly-miR396b were down-regulated in the drought tolerant IL9–1, while they were up-regulated in the sensitive genotype M82. MiR396 and its target *GRF* play important roles in the meristem and leaf development in *Arabidopsis*, and miR396d controls the spikelet development of rice [72–74]. Besides *GRF*, we found that *TDI-65* (Solyc12g088670) could be another target of sly-miR396a-5p and sly-miR396b, which encodes cysteine proteinase. This is also supported by the degradome sequencing in tomato by Karlova et al. [60]. Cysteine proteinase plays important roles in plant stress adaptation through cell establishment, degradation of damaged protein and thus supply of peptides or free amino acids for the biosynthesis of new proteins including stress-related new proteins [75, 76]. It has been demonstrated that cysteine proteinase can be induced by drought stress in many plant species, such as *Arabidopsis*, rice, cotton and poplar [77–80]. This is also the case in tomato [81, 82]. Our RNA-Seq results showed that *TDI-65* was induced at a higher level in IL9–1 than in M82. Our results suggested that miR396a may not only affect tomato growth by targeting *GRF* but also regulate tomato stress tolerance by targeting *TDI-65*.

In addition, 410 target genes were retrieved for the 98 differentially-expressed novel miRNAs induced by drought stress. These genes were involved in cell process, metabolic process, and catalytic activities. A portion of the target genes were believed to be involved in stress response, such as genes encoding transcript factor (MYB, WRKY and bZIP) or receptor-like protein kinase. For instance, sly_miN_780 showed a down-regulation trend both in M82 and IL9–1, but its change in IL9–1 was significant. One target gene of sly_miN_780 was *CIPK6* (Solyc12g010130), which was a potential component of the CBL-CIPK network in calcium signaling. This network has been demonstrated to function in various biotic and abiotic stresses, including high salinity, osmotic stress, drought, cold, ABA and fungi attack [83, 84]. *CIPK6* in *Arabidopsis* is induced by drought and ABA, and overexpression of this gene improves the salt tolerance of *Arabidopsis* plants [85]. Ectopic expression of an apple CIPK, *MdCIPK6L*, in *Arabidopsis*, also confers multiple stress tolerance of the transgenic plants [86]. In our research, sly_miN_780 showed a decreased expression level in the two tomato genotypes tested, in accordingly, the target gene *CIPK6* was up-regulated, which may function in the drought tolerance of tomato. As the change of sly_miN_780 in IL9–1 was significant, it could be a reason of that IL9–1 is more tolerant to drought than M82.

## Conclusions

In this research, we confirmed the superior performance on drought tolerance of the introgression line IL9–1 over its recurrent parent M82, including higher survival rate, higher relative water content, and higher ROS scavenging capability under drought stress condition. Further, deep sequencing was employed to characterize the miRNA and mRNA expression under drought stress for both genotypes. 105 conserved and 179 novel miRNAs were identified, among them, 54 and 98 showed significant changes in expression after stress treatment. A large portion of the predicted target genes of miRNAs were potentially involved in plant stress tolerance, including those encoding transcription factors, drought-stress related proteinase and kinase. RNA-Seq revealed thousands of differentially-expressed genes under drought stress in tomato. Most target genes changed expression in accordance with their miRNAs. GO and pathway analyss on the differentially-expressed genes showed that many genes were involved in stress tolerance, such as those function in plant hormone signal transduction. The better performance of IL9–1 on drought tolerance may be achieved by mobilizing genes to maintain the leaf turgor and to improve ROS elimination. Quite a few miRNAs and their target genes were involved in the regulation of stress-responsive protein, antioxidant enzymes and plant hormone pathways. Overall, our results suggested that miRNAs play essential roles in tomato drought tolerance.

## Methods

### Plant materials and drought treatment

Seeds of the tomato cultivar (M82) and the introgression line IL9–1 were obtained from the Tomato Genetic Resource Center, University of California, Davis, CA, USA. IL9–1 carries a single introgressed segment (0–4,382,155 bp) of chromosome 9 from *S. pennellii* LA0716 [37]*.* A previous screening has identified that IL9–1 is strongly tolerant to drought stress, while M82 is a drought-sensitive recurrent parent [42].

Uniform seeds were geminated under darkness at 28 °C in petri dishes with moist filter papers. Uniformly germinated seeds were sown in plastic pots (size: 7 × 7 × 7 cm) with equivalent dry weight of vermiculite, peat and perlite (1:1:1). The seedlings were grown at 25 ± 2 °C in a growth room with a 16 h/8 h light/dark cycle. Irrigation and fertilizer were applied as needed to ensure healthy growth of the tomato seedlings.

To confirm the drought-tolerant phenotype of IL9–1, tomato seedlings at the five-leaf stage were challenged with drought stress by withdrawing water, while the control plants were irrigated as usual. The experiment was conducted with three replicates per treatment and 10 seedlings for each replicate. All the seedlings were placed randomly on the shelf, and surrounded further by a layer of seedlings to avoid edge effect. For drought treatment, all the pots were soaked in water until full saturation (about 3 cm in depth, and for 12 h), followed by drainage of extra water and a natural dryness. After 10 days of stress, the second leaf from the bottom was sampled for measurement of relative water content. The third leaf from the bottom was sampled, frozen in liquid nitrogen and then stored at −70 °C until use. Three weeks later, recovery was implemented by resupplying water to the pots, and the survival rate was finally recorded.

### Physiological measurements

The relative water content of leaves was measured according to Tuna et al. [87]. The frozen leaf sample was ground into fine powder in liquid nitrogen, and 100 mg of aliquot was used to analyze H_2_O_2_ content. The measurement was conducted according to Huang et al. [88].

### RNA isolation and library preparation

Total RNA was isolated from the above samples using TRIzol reagent (Invitrogen, CA, USA) according to the manufacturer’s instructions. RNA quality was checked by 1% agarose gel electrophoresis, and the concentration was determined by a Nanodrop 2000 spectrophotometer (Thermo Scientific, Wilmington, DE). The RNA samples were sent to Novogene (Beijing, China) to generate libraries for small RNA and mRNA sequencing. RNA integrity and concentration was further checked using the Agilent 2100 Bioanalyzer (Agilent Technologies, CA, USA). The small RNA and mRNA were reverse transcribed to cDNA, and then sequenced with HiSeq 2500 and HiSeq 4000 (Illumina, San Diego, CA, USA), respectively [89].

### Identification of conserved and novel miRNAs

Clean data were obtained by removing adaper of all the reads, reads containing over 10% of N and with low-quality (Q20 < 85%) from the raw data. Potential small RNAs with 16–28 nt in length were extracted, and then mapped to the reference genome of *S. lycopersicum* (http://solgenomics.net, Ver. SL2.5) using bowtie program [90]. Mapped reads were further mapped to Rfam database (http://rfam.xfam.org, version 11.0), and rRNA, tRNA, snRNA and snoRNAs were obtained, respectively [91]. Small RNA reads were kept by removing the reads mapped to the Rfam database, and further mapped to the released tomato miRNAs in miRBase 21 using BLASTN (E-value ≤1e-6) [92]. With these, conserved miRNAs were obtained. After removing the reads already classified into conserved miRNAs, miR-PREFeR (https://github.com/hangelwen/miR-PREFeR) was employed to predict potential novel miRNAs, with permission of 2 bp 3′ overhangs. Putative miRNA with no expression of the STAR sequence and with reads less than 10 were removed to improve the accuracy [93].

To identify the differentially expressed miRNAs, the miRNA expressions in the four samples were normalized to obtain the expression of transcript per million (TPM) on the basis of the normalization formula: normalized expression = (actual miRNA count/total count of mapped reads)*1,000,000. Then, the fold-change, *p*-values and q-values were calculated with in-house perl scripts. The miRNAs with false discovery rate (FDR) -adjusted q-value ≤0.05 and |log2 (fold-change) | ≥ 1 were identified as differentially expressed miRNAs.

### Identification of differentially expressed genes

The quality of RNA-Seq data was evaluated by FastQC software [94]. Clean data was obtained by removing adapter and low-quality reads from the raw data. All clean reads were mapped to the tomato reference genome mentioned above using hisat2 program [95], with the following parameters: --max-intronlen 8000 --dta-cufflinks. The remaining parameters were set as default. All the uniquely mapped reads were extracted and then transformed to sorted bam files with samtools [96]. Fragments per kilobase of exon model per million mapped reads (FPKM) for genes were quantified by Cuffdiff within Cufflinks [97]. After calculating the fold change, genes with FDR-adjusted q-value ≤0.05 and |log2 (fold-change) | ≥ 1 were identified as differentially expressed genes.

### miRNA target prediction

psRNATarget server (http://plantgrn.noble.org/psRNATarget/) was employed to predict the target genes for all the conserved and novel miRNAs that expressed differentially in the two genotypes under drought stress. The parameters in prediction were set as default from the webserver [98].

### GO enrichment, KEGG pathway and network analysis

The online tool agriGO (GO Analysis Toolkit and Database for Agricultural Community) was used in GO enrichment analysis [99]. GO classification was obtained by submitting the gene sequences to the query list of agriGO and selecting locus ID of *S. lycopersium* ITAG2.4. Pathway analysis was based on the KEGG database (http://www.genome.jp/kegg) [100]. Network analysis was conducted using the Cytoscape network platform [101].

### Validation of miRNA and gene expression by qRT-PCR

qRT-PCR was used to validate the deep sequencing results. The reverse transcription and real time PCR were carried out essentially using the Mir-X miRNA qRT-PCR SYBR® Kits (Takara, Dalian, China). Genomic DNA residue was removed by DNase I (Takara) treatment on the total RNA samples. Reverse transcription was conducted in a RNase-free Eppendorf tube, with 10 μl mixture containing 5 μl mRQ buffer (2×), 1.25 μl mRQ enzyme and 8 μg RNA sample. The mixture was incubated at 37 °C for 1 h, followed by inactivation of the reverse transcriptase at 85 °C for 5 min. The resulted cDNA was diluted into 100 μl, and then the products were used as templates to analyze the expression of the miRNAs and genes. Real-time PCR was performed on a LightCycler® 96platform (Roche Diagnostics, Basel, Switzerland), with 25 μl of mixture containing 9.5 μl ddH_2_O, 12.5 μl SYBR Advantage Premix (2×), 0.5 μl of forward and reverse primer (10 μM), and 2 μl cDNA template. All the primers for miRNA and gene amplification were listed in Additional file 18: Table S18. Tomato actin 4 (Solyc04g011500) was used as the internal control. PCR program was set as follow: 95 °C 10 s, 40 cycles of 95 °C 5 s and 60 °C 20 s, followed by a standard melting curve analysis (95 °C 55 s, 55 °C 30 s, 95 °C 30 s). All experiments were repeated three times. The 2^-△△CT^ method was used to calculate the fold change of gene expression, and the expression data were log2 transformed before analysis [102].

## Additional files

Additional file 1: Table S1. Conserved miRNAs expressed in the drought-sensitive and -tolerant tomato genotypes. a, TPM: the expression of transcript per million on the basis of the normalization formula: normalized expression = (actual miRNA count/total count of mapped reads)*1,000,000. b, * and ** indicate a significant difference after drought stress. *: q-value ≤0.05 and |log2 (SD/SCK) | ≥ 1. **: q-value ≤0.01 and |log2 (SD/SCK) | ≥ 1. c, * and ** indicate a significant difference after drought stress. *: q-value ≤0.05 and |log2 (TD/TCK) | ≥ 1. **: q-value ≤0.01 and |log2 (TD/TCK) | ≥ 1. (XLSX 27 kb)

Additional file 2: Table S2. Novel miRNAs expressed in drought-sensitive and -tolerant tomato genotypes. a, TPM: the expression of transcript per million on the basis of the normalization formula: normalized expression = (actual miRNA count/total count of mapped reads)*1,000,000. b, * and ** indicate a significant difference after drought stress. *: q-value ≤0.05 and |log2 (SD/SCK) | ≥ 1. **: q-value ≤0.01 and |log2 (SD/SCK) | ≥ 1. c, * and ** indicate a significant difference after drought stress. *: q-value ≤0.05 and |log2 (TD/TCK) | ≥ 1. **: q-value ≤0.01 and |log2 (TD/TCK) | ≥ 1. (XLSX 34 kb)

Additional file 3: Table S3. List of genes with confident expression in the drought-sensitive tomato M82. a, FPKM: fragments per kilobase of exon model per million mapped reads. (XLSX 1997 kb)

Additional file 4: Table S4. List of genes with confident expression in the drought-tolerant tomato IL9–1. a, FPKM: fragments per kilobase of exon model per million mapped reads. (XLSX 1915 kb)

Additional file 5: Table S5. Significant GO terms among a**.** up-regulated genes under drought in the drought-sensitive tomato M82; b**.** up-regulated genes under drought in the drought-tolerant genotype IL9–1; c**.** down-regulated genes under drought in M82; d**.** down-regulated genes under drought in IL9–1. (DOCX 25 kb)

Additional file 6: Table S6. KEGG pathways of significantly up-regulated genes in the drought-sensitive tomato M82. (XLSX 15 kb)

Additional file 7: Table S7. KEGG pathways of significantly up-regulated genes in the drought-tolerant tomato IL9–1. (XLSX 13 kb)

Additional file 8: Table S8. KEGG pathways of significantly down-regulated genes in the drought-sensitive tomato M82. (XLSX 18 kb)

Additional file 9: Table S9. KEGG pathways of significantly down-regulated genes in the drought-tolerant tomato IL9–1. (XLSX 14 kb)

Additional file 10: Table S10. Target gene prediction results of the differentially-expressed conserved miRNAs. (XLSX 56 kb)

Additional file 11: Table S11. Enriched GO terms of the target genes for the differentially-expressed conserved miRNAs in tomato. a, Number in input list: the number of genes mapped to the GO term of input list. b, Number in BG/Ref: the number of genes mapped to the GO term of reference list. (XLSX 13 kb)

Additional file 12: Table S12. KEGG pathways of the target genes for the differentially-expressed conserved miRNAs in tomato. (XLSX 10 kb)

Additional file 13: Table S13. Expression level of the target genes for the differentially-expressed conserved miRNAs identified. (XLSX 29 kb)

Additional file 14: Table S14. Target gene prediction results of the differentially-expressed novel miRNAs. (XLSX 118 kb)

Additional file 15: Table S15. Enriched GO terms of the target genes for the differentially-expressed novel miRNAs in tomato. a, Number in input list: the number of genes mapped to the GO term of input list. b, Number in BG/Ref: the number of genes mapped to the GO term of reference list. (XLSX 14 kb)

Additional file 16: Table S16. KEGG pathways of the target genes for the differentially-expressed novel miRNAs in tomato. (XLSX 12 kb)

Additional file 17: Table S17. Expression level of the target genes for the differentially-expressed novel miRNAs identified. (XLSX 45 kb)

Additional file 18: Table S18. The primers of miRNAs and genes used for qRT-PCR verification (XLSX 10 kb)

## Abbreviations

GO: Gene ontology
KEGG: Kyoto Encyclopedia of Genes and Genomes
miRNAs: microRNAs
nt: Nucleotides
qRT-PCR: quantitative real-time PCR
