# Supplementary material for: Profiling of drought-responsive microRNA and mRNA in tomato using high-throughput sequencing
Source: BMC Genomics. 2017 Jun 26;18:481. doi: 10.1186/s12864-017-3869-1 (PMC5485680; doi:10.1186/s12864-017-3869-1)
Supplement: Supplementary file 5 — Significant GO terms among a. up-regulated genes under drought in the drought-sensitive tomato M82; b. up-regulated genes under drought in the drought-tolerant genotype IL9–1; c. down-regulated genes under drought in M82; d. down-regulated genes under drought in IL9–1. (DOCX 25 kb) [file 12864_2017_3869_MOESM5_ESM.docx]

**Additional file 5: Table S5 -** Significant GO terms among **a.** up-regulated genes under drought in the drought-sensitive tomato M82; **b.** up-regulated genes under drought in the drought-tolerant genotype IL9-1; **c.** down-regulated genes under drought in M82; **d.** down-regulated genes under drought in IL9-1.

**Supplementary Table 5a**

| GO term | Ontology | Description | p-value |
| --- | --- | --- | --- |
| GO:0009415 | P | response to water | 2.20E-05 |
| GO:0042221 | P | response to chemical stimulus | 4.60E-05 |
| GO:0051082 | F | unfolded protein binding | 5.70E-06 |
| GO:0016762 | F | xyloglucan: xyloglucosyl transferase activity | 3.20E-05 |

**Supplementary Table 5b**

| GO term | Ontology | Description | p-value |
| --- | --- | --- | --- |
| GO:0009415 | P | response to water | 4.50E-06 |
| GO:0042221 | P | response to chemical stimulus | 8.50E-06 |
| GO:0009628 | P | response to abiotic stimulus | 6.10E-05 |
| GO:0051213 | F | dioxygenase activity | 0.00049 |
| GO:0003677 | F | DNA binding | 8.90E-05 |
| GO:0043565 | F | sequence-specific DNA binding | 0.00022 |
| GO:0016702 | F | oxidoreductase activity, acting on single donors with incorporation of molecular oxygen, incorporation of two atoms of oxygen | 0.00029 |
| GO:0051082 | F | unfolded protein binding | 0.0005 |
| GO:0016701 | F | oxidoreductase activity, acting on single donors with incorporation of molecular oxygen | 0.00043 |

**Supplementary Table 5c**

| GO term | Ontology | Description | p-value |
| --- | --- | --- | --- |
| GO:0071555 | P | cell wall organization | 9.00E-06 |
| GO:0071669 | P | plant-type cell wall organization or biogenesis | 9.00E-06 |
| GO:0009664 | P | plant-type cell wall organization | 9.00E-06 |
| GO:0071554 | P | cell wall organization or biogenesis | 0.00015 |
| GO:0003824 | F | catalytic activity | 8.00E-15 |
| GO:0004675 | F | transmembrane receptor protein serine/threonine kinase activity | 2.40E-14 |
| GO:0004702 | F | receptor signaling protein serine/threonine kinase activity | 9.40E-14 |
| GO:0005057 | F | receptor signaling protein activity | 2.10E-13 |
| GO:0004674 | F | protein serine/threonine kinase activity | 3.20E-12 |
| GO:0004872 | F | receptor activity | 7.20E-11 |
| GO:0004888 | F | transmembrane receptor activity | 3.30E-10 |
| GO:0004672 | F | protein kinase activity | 3.00E-10 |
| GO:0060089 | F | molecular transducer activity | 4.60E-10 |
| GO:0004871 | F | signal transducer activity | 4.60E-10 |
| GO:0019199 | F | transmembrane receptor protein kinase activity | 4.40E-10 |
| GO:0016740 | F | transferase activity | 5.00E-10 |
| GO:0016773 | F | phosphotransferase activity, alcohol group as acceptor | 1.10E-09 |
| GO:0016168 | F | chlorophyll binding | 4.60E-09 |
| GO:0016772 | F | transferase activity, transferring phosphorus-containing groups | 7.00E-09 |
| GO:0008236 | F | serine-type peptidase activity | 1.50E-08 |
| GO:0005372 | F | water transmembrane transporter activity | 1.80E-08 |
| GO:0015250 | F | water channel activity | 1.80E-08 |
| GO:0017171 | F | serine hydrolase activity | 2.50E-08 |
| GO:0016301 | F | kinase activity | 3.40E-08 |
| GO:0004252 | F | serine-type endopeptidase activity | 3.00E-07 |
| GO:0022838 | F | substrate-specific channel activity | 2.10E-06 |
| GO:0022803 | F | passive transmembrane transporter activity | 3.20E-06 |
| GO:0015267 | F | channel activity | 3.20E-06 |
| GO:0046906 | F | tetrapyrrole binding | 4.80E-06 |
| GO:0004175 | F | endopeptidase activity | 1.50E-05 |
| GO:0022892 | F | substrate-specific transporter activity | 4.60E-05 |
| GO:0016614 | F | oxidoreductase activity, acting on CH-OH group of donors | 5.40E-05 |
| GO:0016759 | F | cellulose synthase activity | 5.40E-05 |
| GO:0016798 | F | hydrolase activity, acting on glycosyl bonds | 6.60E-05 |
| GO:0016491 | F | oxidoreductase activity | 8.00E-05 |
| GO:0022891 | F | substrate-specific transmembrane transporter activity | 9.00E-05 |
| GO:0008810 | F | cellulase activity | 0.00034 |
| GO:0016616 | F | oxidoreductase activity, acting on the CH-OH group of donors, NAD or NADP as acceptor | 0.00034 |
| GO:0016758 | F | transferase activity, transferring hexosyl groups | 0.00036 |
| GO:0070011 | F | peptidase activity, acting on L-amino acid peptides | 0.00044 |
| GO:0046527 | F | glucosyltransferase activity | 0.00052 |
| GO:0022857 | F | transmembrane transporter activity | 0.00069 |
| GO:0008233 | F | peptidase activity | 0.00095 |
| GO:0005215 | F | transporter activity | 0.001 |
| GO:0034357 | C | photosynthetic membrane | 2.60E-06 |
| GO:0009538 | C | photosystem I reaction center | 5.10E-06 |
| GO:0009521 | C | photosystem | 3.60E-06 |
| GO:0009579 | C | thylakoid | 5.70E-06 |
| GO:0009522 | C | photosystem I | 4.30E-05 |

**Supplementary Table 5d**

| GO term | Ontology | Description | p-value |
| --- | --- | --- | --- |
| GO:0071555 | P | cell wall organization | 2.90E-06 |
| GO:0071669 | P | plant-type cell wall organization or biogenesis | 2.90E-06 |
| GO:0009664 | P | plant-type cell wall organization | 2.90E-06 |
| GO:0071554 | P | cell wall organization or biogenesis | 0.0011 |
| GO:0016168 | F | chlorophyll binding | 3.10E-10 |
| GO:0003824 | F | catalytic activity | 1.20E-09 |
| GO:0046906 | F | tetrapyrrole binding | 6.70E-08 |
| GO:0016798 | F | hydrolase activity, acting on glycosyl bonds | 7.50E-08 |
| GO:0004675 | F | transmembrane receptor protein serine/threonine kinase activity | 2.00E-07 |
| GO:0004252 | F | serine-type endopeptidase activity | 3.50E-07 |
| GO:0008236 | F | serine-type peptidase activity | 6.80E-07 |
| GO:0017171 | F | serine hydrolase activity | 9.30E-07 |
| GO:0004702 | F | receptor signaling protein serine/threonine kinase activity | 1.70E-06 |
| GO:0005057 | F | receptor signaling protein activity | 2.50E-06 |
| GO:0005372 | F | water transmembrane transporter activity | 4.40E-06 |
| GO:0015250 | F | water channel activity | 4.40E-06 |
| GO:0004175 | F | endopeptidase activity | 4.80E-06 |
| GO:0004553 | F | hydrolase activity, hydrolyzing O-glycosyl compounds | 6.30E-06 |
| GO:0004888 | F | transmembrane receptor activity | 1.30E-05 |
| GO:0016787 | F | hydrolase activity | 1.50E-05 |
| GO:0004872 | F | receptor activity | 1.70E-05 |
| GO:0019199 | F | transmembrane receptor protein kinase activity | 2.00E-05 |
| GO:0004091 | F | carboxylesterase activity | 3.00E-05 |
| GO:0004674 | F | protein serine/threonine kinase activity | 3.60E-05 |
| GO:0022838 | F | substrate-specific channel activity | 0.00013 |
| GO:0070011 | F | peptidase activity, acting on L-amino acid peptides | 0.00013 |
| GO:0060089 | F | molecular transducer activity | 0.00016 |
| GO:0022803 | F | passive transmembrane transporter activity | 0.00017 |
| GO:0015267 | F | channel activity | 0.00017 |
| GO:0004871 | F | signal transducer activity | 0.00016 |
| GO:0008810 | F | cellulase activity | 0.00021 |
| GO:0008233 | F | peptidase activity | 0.00024 |
| GO:0004672 | F | protein kinase activity | 0.00051 |
| GO:0016614 | F | oxidoreductase activity, acting on CH-OH group of donors | 0.0008 |
| GO:0004866 | F | endopeptidase inhibitor activity | 0.0022 |
| GO:0030599 | F | pectinesterase activity | 0.0021 |
| GO:0016773 | F | phosphotransferase activity, alcohol group as acceptor | 0.0026 |
| GO:0016491 | F | oxidoreductase activity | 0.0026 |
| GO:0016616 | F | oxidoreductase activity, acting on the CH-OH group of donors, NAD or NADP as acceptor | 0.0027 |
| GO:0009538 | C | photosystem I reaction center | 2.60E-06 |
| GO:0009521 | C | photosystem | 1.40E-06 |
| GO:0034357 | C | photosynthetic membrane | 1.30E-05 |
| GO:0009522 | C | photosystem I | 1.60E-05 |
| GO:0009579 | C | thylakoid | 2.20E-05 |
| GO:0005618 | C | cell wall | 0.0023 |
